# A Nursing and Computer Science Perspective on Confronting Chronic Illness and Environmental Responsibility in AI Research

**DOI:** 10.3390/nursrep16030094

**Published:** 2026-03-09

**Authors:** S. Raquel Ramos, Rex Ying

**Affiliations:** 1 School of Nursing, Yale University, Orange, CT 06477, USA; 2 Department of Social and Behavioral Sciences, School of Public Health, Yale University, New Haven, CT 06520, USA; 3 Center for Interdisciplinary Research on AIDS, New Haven, CT 06520, USA; 4 Department of Computer Science, School of Engineering, Yale University, New Haven, CT 06520, USA; rex.ying@yale.edu

**Keywords:** artificial intelligence, large language models, interdisciplinary teams, CVD, patient education, chronic disease, environmental health, natural language processing, sustainability, nursing innovation

The exponential growth of artificial intelligence has transformed global information ecosystems, introducing complex technological challenges that extend far beyond its computational capabilities. In nursing, generative AI and large language models are rapidly reshaping how clinicians assess patients, predict risk, personalize care plans, and coordinate services. These tools offer new ways to envision and revolutionize health care to optimize patient care and improve health outcomes. While unprecedented in potential, this technological revolution also carries complex environmental and public health implications, which warrant attention as nurses confront rising chronic disease burdens and cardiovascular risk factors.

Cardiovascular risk factors are rising and current US data show a substantial chronic disease burden through to 2050 [1]. In 2023, about 76.4% of U.S. adults reported at least one chronic condition and 51.4% experience multimorbidity [2]. Obesity affects 40.3% of adults, hypertension affects 47.3%, type 2 diabetes affects 13.5%, and prediabetes affects 37.2% [1]. These statistics demonstrate the serious population-level impact of cardiovascular illness and call for greater emphasis on prevention, self-management, and community-based care.

To address this issue, a nurse-led interdisciplinary research team composed of experts in nursing, computer science, public health, and medicine developed CARDIO [3]: a fine-tuned, large language model designed to support cardiovascular disease prevention in clinical settings. CARDIO was conceived as a complementary tool to enhance, rather than replace, clinician expertise. Development prioritized patient education, health literacy, and workflow integration so that the tool would support clinicians without adding burden. These nursing priorities also prompted attention to AI’s environmental and public health implications. Linking clinical priorities with system stewardship, our team treated environmental and public health impacts as integral design considerations.

We strategically fine-tuned a compact Llama base model, curating authoritative clinical sources and patient forum content to create domain-specific training material that prioritized precision and efficiency [3]. During development, we also assessed AI’s broader systemic impacts beyond computation, including the environmental burdens of data center infrastructure. Currently, the United States hosts 3891 data centers nationwide, and the planned expansions could substantially increase national power needs [4]. Projected expansions suggest a potential 1000% increase in computing capacity, with over 150 gigawatts of new power capacity planned [5]. The water consumption required for AI model training has been estimated to be as high as 700,000 L for some large models, contributing to a projected increase in electricity consumption from 4.4% to 12% nationally [5]. These figures quantify the profound environmental ramifications of data centers that directly impact the population’s physiological vulnerabilities. These estimates suggest that environmental factors associated with data center infrastructure may undermine individuals’ health, leading to a greater risk of preventable chronic illnesses. For nursing practice, this means that AI’s energy footprint is an important clinical concern and should inform model design deployment decisions.

In response to these challenges, our interdisciplinary team offers focused recommendations for responsible AI in nursing research and clinical practice. First, we prioritize rule-based models for tasks that do not require large language models, because when nurses conceptualize or implement AI tools in research or clinical care, they should start with the most efficient approach that meets the clinical need. Second, in practice, nurses innovating with AI should favor lightweight, task-specific architectures and targeted fine-tuning of compact models to minimize resource consumption, preserve interpretability, and evaluate usability before wider deployment. Third, nurse researchers and clinical informaticists should build decommissioning criteria into governance timelines so that outdated or unused models are retired and unnecessary energy use is avoided. Finally, nurse researchers and clinicians should advocate for regulatory and procurement standards that link clinical safety and sustainability so that nursing expertise can guide responsible development and deployment within institutions and professional organizations, and practicing nurses should provide case examples and outcome data to ensure that the standards reflect the frontline realities.

The future of technological development must transcend mere computational capabilities to embrace holistic human and environmental wellness. Our approach with CARDIO demonstrates that AI can be simultaneously clinically sophisticated and environmentally responsible. We leverage a light-weight, fine-tuned model that was designed to complement clinical expertise without imposing unsustainable computational burdens. Interdisciplinary collaboration was essential for reimagining technological infrastructure as integrated ecological participants. By centering clinical requirements, implementing careful design, executing targeted fine-tuning, and maintaining operational transparency, nurses can develop AI technologies that enhance patient education, conserve critical resources, and preserve clinician time for high-value interventions. CARDIO provides a concrete example of this approach in practice, illustrating the potential for AI to contribute positively to both nursing care and environmental sustainability.

## Abbreviations

The following abbreviations are used in this manuscript:

AI	Artificial Intelligence
CARDIO	Conversational AI for Reducing Disparities and Improving Outcomes
CVD	Cardiovascular Disease
LLM	Large Language Model
